# Cross-cultural training program on mental health care for refugees - a mixed method evaluation

**DOI:** 10.1186/s12909-021-02965-5

**Published:** 2021-10-15

**Authors:** Jordanos Tewelde McDonald, Marie Dahlin, Sofie Bäärnhielm

**Affiliations:** 1 Department of Global Public Health, Karolinska Institute & Transcultural Centre, Region Stockholm, Solnavägen 4, 11365 Stockholm, Sweden; 2 Department of Clinical Neuroscience, Centre for Psychiatry Research, Karolinska Institutet & Stockholm Health Care Services, Region Stockholm, Norra Stockholms psykiatri, Vårdvägen 1, 11281 Stockholm, Sweden; 3 Department of Clinical Neuroscience, Centre for Psychiatry Research, Karolinska Institutet, & Stockholm Health Care Services, Region Stockholm, Transcultural Centre, Region Stockholm, Solnavägen 4, 11365 Stockholm, Sweden

**Keywords:** Cross-cultural training, Mental health care, Refugees, Asylum seekers

## Abstract

**Background:**

Refugees tend to have a higher risk of mental ill-health and use mental health services less than the native-born population during their first 10 years in Sweden. Intercultural interactions between refugees and mental health professionals have been described as challenging. Cross-cultural training is proposed as one way to improve care for refugees. Evaluations of such training outcomes are sparse. The overall aim of this study was to evaluate Comprehensive Cross-Cultural Training for mental health care professionals in Stockholm, and to assess training outcomes for participants’ perceived knowledge regarding mental health and care for newly arrived refugee patients, asylum seekers and undocumented refugees. In addition, we analysed the dimensionality of the pre- and post-training questionnaires used.

**Methods:**

An embedded mixed-method design was applied. We used pre-and post-training questionnaires (*n* = 248) and conducted six focus group discussions (FGDs) with mental health professionals after training. Quantitative data was analysed by t-tests and factor analysis, qualitative data was analysed using thematic content analysis.

**Results:**

Participants experienced gained knowledge and new perspectives in all aspects covered in the training. Training led to participants restructuring their existing knowledge. Those who had reported experience of refugee patients and working with interpreters pre-training in the past month, had higher ratings of perceived knowledge. Post-training, there were no significant changes in perceived knowledge between those with, and those without, experience of refugee patients and working with interpreters. Factor analysis resulted in 3 factors for the pre-training questionnaire, explaining 71% of the covariance, and 4 factors for the post-training questionnaire, explaining 78% of the covariance. Findings from the post-training FGDs, revealed that refugee patients were described as challenging. Also, that training promoted empathy towards refugees and strengthened participants’ professional role.

**Conclusions:**

This cross-cultural training contributed to knowledge development and attitude changes. It was valuable regarding care providers’ professional role. Additional outcomes of the training were that participants not only gained knowledge about refugee mental health care but also restructured their existing knowledge.

**Supplementary Information:**

The online version contains supplementary material available at 10.1186/s12909-021-02965-5.

## Background

Given that refugees tend to have a higher risk of mental ill-health [1], along with lower mental health care utilization in Europe [2, 3], there is an urgent need of improvement of mental health services. Intercultural communication and interaction between health care professionals and refugee patients have been described as challenging [4–6] and require knowledge and skills among professionals. Knowledge among professionals makes cross-cultural training for mental health care providers a key factor in improving care for refugees [5–7].

The migration process influences the health of migrants, in particular refugees and asylum seekers [1–8]. Prevalence of mental disorders such as depression, anxiety, post-traumatic stress, and psychosis is higher among refugees than other migrants and natives [1–3, 7, 9–12]. A Swedish study showed that approximately 33% of the newly arrived refugees from Syria and asylum seekers from Eritrea and Somalia had mental health problems, such as depression and anxiety [13]. Despite the likelihood of higher psychopathology among refugee groups, there are European data suggesting that refugees use less mental health services than the native population [2, 3, 7, 11, 14]. In Sweden, utilization of psychiatric care among migrants is lower than for the Swedish-born population during the ten first years, apart from compulsory care where care consumption is higher [15, 16]. Barriers to mental health care among migrants are seen both on the individual and structural level [1, 2, 5, 8, 11, 12, 14, 15, 17, 18]. Further, barriers both from the perspective of refugees and asylum seekers, as well as from service providers, may contribute to the lower use of mental health care. Regarding refugees and asylum seekers, they face barriers related to language problems, stigma, lack of information about mental health care and lack of cultural sensitivity in the mental health care services [1, 2, 5, 8, 11, 12, 14, 15, 17, 18]. Barriers facing the service providers are linked to lack of competency in intercultural communication [6] and working with cultural variety among this group of patients [5, 6, 17, 18]. Swedish mental health care professionals’ understanding of rights to care for asylum seekers’ and undocumented refugees may be additional barriers [19].

### Mental health care for asylum seekers and refugees in Stockholm

Sweden has received a great number of refugees and asylum seekers, creating challenges for mental health care to adapt to the need of the newcomers. Between 2000 and 2018, Sweden received 733,827 asylum seekers [20] with a peak in 2015, when approximately 164,000 were seeking asylum. Between 2015 and June 2019, 106,287 unaccompanied minors arrived [20]. Most of the refugees came from Syria, Eritrea, Iraq, Somalia and Afghanistan, countries affected by war, conflict or economic crises [8, 13, 20–22].

Region Stockholm is responsible for the health care of all its residents, amounting to approximately 2 million people, one fifth of the Swedish population. The mental health care system mainly includes tax-funded public health care providers, although there are privately- funded providers within Region Stockholm [10]. Migrants and refugees with permission to stay are entitled to health care under the same conditions as any Swedish resident. The same holds for children and young people up to 18 years who are asylum seekers or undocumented migrants [10]. However, adult asylum seekers and undocumented migrants can only receive “care that cannot be deferred” [10, 19] and the evaluation of whether care is needed is determined by the physician encountering the patient.

To understand the right to care for asylum seekers and undocumented refugees, mental health professionals ought to be updated in legislation and regulations [19]. In order to provide high quality mental health care for these groups and newly arrived refugees with a residence permit, mental health care professionals need knowledge in areas such as: interpretation of cultural variety in expressions of distress, the importance of culture and context for diagnostics and treatment in a clinical setting, identification of symptoms of PTSD and how to make effective use of interpreters [5, 7, 11]. Training health care providers has been suggested as one way to improve access and quality of mental health care for refugees [3, 5–7, 9]. In a Swedish study based in child health care centres, cross-cultural training supported nurses in their clinical work – in particular, in providing culturally sensitive health services. After training, the nurses assessed themselves as being more confident in their encounters with refugee patients [6]. Another study evaluating a basic training program in transcultural psychiatry, given to health care professionals, found that after the training program participants experienced increased knowledge and an improved ability in meeting refugee patients with mental disorders [23].

There are relatively few studies on the outcomes of cross-cultural training interventions and how the training affects cross-cultural knowledge among staff. Due to the limited research and evaluations conducted on cross-cultural training, more research initiatives are needed to address how these interventions can be implemented efficiently, and which training efforts are effective [23, 24].

### Aim

The overall aim of this study was to evaluate Comprehensive Cross-Cultural Training (CCCT) for mental health care professionals in Stockholm. The specific aims were to evaluate training outcome on: 1) participants’ perceived knowledge and skills regarding mental health and care for newly arrived refugee patients, asylum seekers, and undocumented refugees, 2) participants’ perceived knowledge and skills after training, and 3) whether any changes were related to participants’ recent experiences working with these groups of people. Lastly, we wanted to evaluate any possible difference in the dimensionality of the pre-and post-training questionnaires as a result of the training experience.

## Method

### Comprehensive cross-cultural training

Comprehensive Cross-cultural Training (CCCT) was initiated in 2016 to respond to the mental health care challenges at a time of high influx of asylum seekers and refugees in Region Stockholm. The CCCT targeted psychiatric care providers and was divided into introductory and advanced programs. This evaluation focusses on the introductory training. The program was organized by the Transcultural Centre (TC), a knowledge centre in the field of transcultural psychiatry and migration and health in Region Stockholm. The TC has long experience of training health and mental health professionals. The CCCT intended to build and improve knowledge as well as the capacity of psychiatry care providers in Region Stockholm to address mental health needs of refugees, asylum seekers and undocumented migrants, both adults and minors. Planning of the training program was made by the TC in collaboration with a steering group of managers from the major psychiatry organizations in Stockholm and a reference group including professionals working at the various clinics and organizations.

CCCT-educators included psychiatrists, psychologists, nurses and social workers from Region Stockholm. The content of the training was based on previous training experiences at the TC and needs were formulated by the steering and the reference groups based on a review of literature of research of cross-cultural training of mental health care professionals [23, 24]. The literature review involved previously conducted cross-cultural training in Stockholm for participants working in refugee reception within the local social services, in mental health care (primary care and psychiatric care) and in employment agencies [23, 24]. Unlike earlier evaluation, the CCCT targeted current clinical challenges for professionals working in psychiatry and had less focus on primarily enhancing contact between different agencies regarding refugee reception within the local social services and the role of various agencies for refugee receptions and care [23, 24].

The precise content of the CCCT included lectures covering: regulations and authorities involved in refugee reception; rights and access to care for migrants with different types of civic status (asylum seekers, undocumented and refugees with a residence permit); how the migration process and trauma may affect refugee health; culture and psychopathology; working with interpreters and talking about trauma, torture and migration with patients.

The CCCT involved interactive lectures, discussions, and case presentations in large groups in Swedish. Participants were given time and opportunities to interact with lecturers and each other in terms of asking questions, reflecting on case presentations, sharing experiences and discussions. The range of participants at each training intervention was 15–40 persons.

### Mixed-method evaluation

To evaluate the CCCT, an embedded mixed-method design was chosen, using questionnaires for quantitative purposes and focus group discussions (FGD) for qualitative purposes. A mixed method embedded design is used when an intervention is conducted and the main measurements evaluating it are quantitative data. Qualitative data are used to enhance, explain, and support the quantitative data, before, during or after the intervention is completed [25]. The design was used to help to understand how participants experienced the training interventions, to better understand the training outcome and determine why the program worked or not [26]. This study had a concurrent design, i.e. quantitative and qualitative data were collected simultaneously during the course of the CCCT day [27].

The quantitative research questions were: had training participants’ self-rated knowledge and skills levels changed after the training and if so, were changes related to their recent experiences? The qualitative research question was: what were training participants’ perceptions and experiences of the CCCT, as well as their views of delivering mental health services to newly arrived refugees after the training? The mixed-method research question was: How has training affected participants’ perceived knowledge and attitudes regarding mental health and care for newly arrived refugee patients, asylum seekers, and undocumented refugees?

### Study population and sampling

**Fig. 1:**
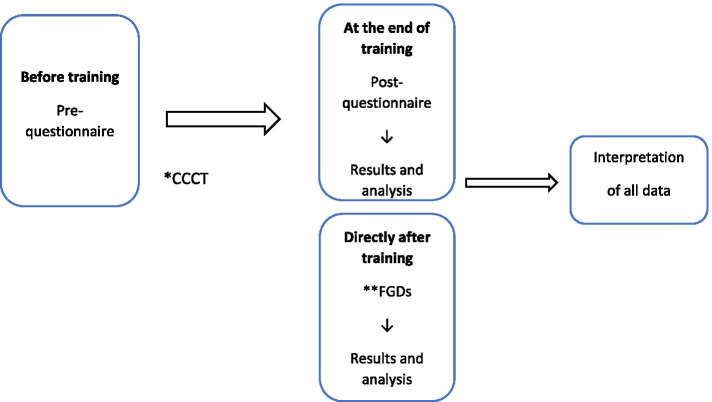
Flow-chart of data collection with a mixed-method embedded design. * CCCT- Comprehensive Cross-Cultural Training. ** FGDs- Focus group discussions

All mental health care professionals in Stockholm were eligible to enrol in the CCCT, through the TC website, and information packages, including study aim and procedures that were sent to all mental health care providers in Stockholm. A total of 248 training participants enrolled in the CCCT. These were primarily psychologists, psychiatrists, psychotherapists, social workers, nurses, nurses aide or administrative staff from mental health services. Besides psychiatry staff, some other participants were from primary care and social and employment services in Stockholm (See Table 1). This study had a nested relationship sample, where FGD informants represented a subset of all CCCT participants (see Fig. 1). Heterogeneous purposive sampling was used for the FGDs. The first author, JTM, presented the study at the beginning of each CCCT and offered training participants the opportunity to take part in an FGD at the end of the training day. In total, 28 informants participated, each FGD included 4–7 participants. Descriptive data of the 28 informants are summarized in Table 4.

**Table 1:** Descriptive data of participants enrolled in the CCCT (*N* = 248)

Item		N (%)
Organization	Adult psychiatry care	154(62.0%)
	Child and adolescent psychiatry care	46(18.5%)
	Addiction care	5(2.0%)
	Other	42(16.9%)
	Missing	1(0.4%)
Occupation/employment	Medical doctors/med. Students	15 (6.0%) (2 medical students)
	Social worker	39 (15.7%)
	Psychologist/psychotherapist	76 (30.6%)
	Nurses	53 (21.3%)
	Auxiliary/psychiatric nurse aide	21 (8,5%)
	Occupational therapist	5 (2%)
	Physiotherapist	4(1.6%)
	Midwife	4(1.6%)
	Management	7(2.8%)
	Administrative staff	7(2.8%)
	Missing	14 (5.6%)
Frequency of professional encounters with refugees^a^ in the past month	0	87 (35%)
	1–3	71 (28.6%)
	3–5	26 (10.5%)
	>5	44 (17.7%)
	Missing	20 (8.0%)
Frequency of using interpreters in the past month	0	90 (36.3%)
	1–3	76 (30.6%)
	4–10	41 (16.5%)
	>10	22 (8.9%)
	Missing	19 (7.7%)
Attitudes towards working with refugees in comparison with non-refugee patients	More difficult	148 (59.7%)
	The same	27 (10.9%)
	Easier	4 (1.6%)
	Missing	69 (27.8%)
Previously attended training about migration, mental ill-health and trauma	Yes	86 (34.7%)
	No	144 (58.1%)
	Missing	18 (7.3%)

^a^including asylum seekers and undocumented migrants

### Data collection

The CCCT interventions took place during the period September 2016–November 2018. In total, 13 introductory training interventions were conducted, 12 one-full day training interventions and 2 half-day interventions. At each training intervention, all participants completed pre- and post-training questionnaires. JTM collected all data by approaching training participants prior to the start of the training and collected all questionnaires at the end of the day.

Prior to the training session, information e-mails including study aim and ethical aspects were sent to all participants. In the questionnaires and before participation in an FGD, all participants/informants were informed that participation was voluntary and anonymous. Furthermore, they were also informed that they had the right to withdraw at any time without giving any reason. At the start of each training, JTM described the questionnaires, and that by completing the questionnaire, they agreed that data obtained would be used and published under anonymity. After the oral presentation at the start of the training program and before each FGD, the participants in agreement were asked to give their informed consent verbally. All participants gave an informed consent verbally to JTM.None of the participants were under the age of 18. Informants in the FGDs received two cinema tickets. All quotes were anonymized.

### Questionnaires

Quantitative data were based on two structured questionnaires, the pre- and post-training questionnaires. For descriptive purposes, the pre-training questionnaires included questions about participants’ experience of clinical work, such as frequency of encounters with refugee patients (including asylum seekers and undocumented migrants) as well as use of interpreters in the past month, rated on a 4-point scale. There were also questions about their attitudes towards working with refugee groups in comparison with non-refugee groups, rated on a 3-point scale, and whether they had attended training in cross-cultural mental health care prior to the CCCT (see Table 1). In the post-training questionnaire, participants were also asked to rate the level and assumed usefulness of the training, and to give their views of their capacity of caring for refugee patients (including asylum seekers and undocumented migrants) in their clinical practice, rated on a 5-point scale.

To evaluate the effect of the training, pre-and post-training questionnaires included 17 identical statements covering participants’ perceived knowledge and skills. They were modified from previous questionnaires, used in an educational evaluation in Stockholm, to adapt to the current training. Changes mainly concerned questions on collaboration and the role of various agencies [23]. The pre-and post questionnairescovered: participants’ perceived knowledge of regulations and authorities involved in refugee reception; rights and access to care for migrants with various civic status (asylum seekers, undocumented and refugees with a residence permit); how the migration process and trauma may affect refugee health; culture and psychopathology; working with interpreters and talking about trauma, torture and migration with patients. Each item was rated on a 5- point Likert scale described as, 1= “completely insufficient for my work” to, 5 = “completely sufficient for my work”. The mean values of the 17 items ranged from 17 to 85 (see Table 2).

**Table 2:** Perceived knowledge for pre- and post-training items and paired t-test (*N* = 192)

Items (Questionnaire) Perceived knowledge about:	(N)	Pre CCC-training Mean (SD)	Post CCC-training Mean (SD)	Paired t-test t-value df	*p*-value
1. Regulations for applying for asylum	181	2.35 (1.06)	3.30 (0.98)	− 11.99180	p < 0.001
2. Asylum seekers’ rights to health care	187	2.67 (1.04)	4.11 (0.84)	−18.60186	p < 0.001
3. Undocumented rights to healthcare	185	2.49 (1.02)	4.06 (0.86)	− 20.20184	*p* < 0.001
4. Refugees’, with residence permits, rights to health care	183	2.87 (1.17)	4.16 (0.82)	−15.80182	*p* < 0.001
5. What actions are being taken for refugees to facilitate integration during their first 2 years in Sweden	182	2.26 (0.98)	3.44 (0.93)	−15.15181	p < 0.001
6. Which authorities are responsible for various actions	185	2.43 (1.04)	3.47 (0.96)	−13.57184	*p* < 0.001
7. How migration process can affect children’s mental health	184	2.92 (1.05)	4.04 (0.80)	−15.18183	*p* < 0.001
8. How migration process can affect adults’ mental health	188	2.87 (0.98)	4.08 (0.77)	−16.33187	p < 0.001
9.How migration process can affect the family situation	184	2.85 (1.05)	3.98 (0.77)	−15.47183	p < 0.001
10.How severe trauma can affect health	163	3.53 (0.99)	4.18 (0.74)	−9.33162	*p* < 0.001
11.How to interpret symptoms of mental illness among asylum seekers and newly arrived refugees	164	2.73 (0.95)	3.69 (0.90)	−13.72163	p < 0.001
12. Which treatment initiatives for newly arrived refugees are various health care providers responsible for	180	2.23 (1.12)	3.45 (0.90)	−14.20179	p < 0.001
13.How to work with an interpreter	174	3.36 (1.12)	4.12 (0.70)	−9.27173	p < 0.001
How to encounter refugees regarding					
14.Severe trauma	182	2.68 (1.16)	3.50 (0.98)	−11.76181	p < 0.001
15.Experience of torture	181	2.45 (1.12)	3.35 (1.05)	− 11.90180	p < 0.001
16.Experience of flight experiences	181	2.61 (1.16)	3.50 (0.90)	− 11.65180	p < 0.001
17. Experience of coming to Sweden	179	2.68 1.13	3.55 (0.94)	− 11.28178	p < 0.001

### Focus group discussions

The participants’ experience and views of delivering mental health care to refugees, as well as how they evaluated the CCCT interventions, were explored in the FGDs. The first author JTM moderated all FGDs. At 4 out of 6 FGDs there was an observer present. The role of the observer was to take notes and assist with the recording. After each FGD, the moderator and the observer together reflected over the content and group dynamics during the FGD.

Six FGDs were conducted directly after training sessions. The semi-structured interview guide included questions about their experience of meeting asylum seekers and refugee patients, attitudes to, and perceptions of, the CCCT and perceived knowledge and skill gains to be able to encounter these patients after the CCCT. During the FGDs, the participants were asked about the content, level and their general views of the different parts of the introductory CCCT. They were also asked about the need for further training. Having adopted purposive sampling, saturation was reached in terms of informants’ experience of the training program and encountering refugee patients in their clinical setting. As described above, after six focus group discussions, no new information was forthcoming and therefore, the information obtained was thus judged as sufficient.

### Statistical analysis

Quantitative data was analysed using SPSS 24. Comparison of means of the pre-and post-ratings of each of the knowledge questions was conducted by paired t-tests (Table 2). Sub-groups were created and dichotomized by participants’ recent experiences/ no experience of encountering refugee patients and of using interpreters. These sub-groups were then compared for change in perceived knowledge pre-and post-training by a t-test for independent groups. For those comparisons we computed mean values and SD of the total score for the 17 items. Finally, in order to evaluate possible differences in dimensionality of the questionnaires, based on the experience of having participated in the training, we performed a factor analysis with Promax rotation and Kaiser normalization of the 17-items on pre-and post-training. Results were presented as mean values and SD for each factor pre-and post-training intervention, along with Cronbach’s alpha for each factor.

### Qualitative data analysis

All the FGDs were conducted by JTM, audio-taped and lasted for about 30–60 min. A research assistant transcribed all the FGDs verbatim. Analysis of the qualitative data was conducted with a thematic content analysis (TCA) [28], with the support of NVivo 11 software [29]. FGDs were analysed parallel to data collection. The analysis process started with JTM and the last author SB, separately listening to the FGD recordings and reading and re-reading the transcripts. The procedure of qualitative analysis involved first identifying meaning units, then condensing them, coding, identifying categories and finally, themes [28]. The qualitative data analysis was primarily performed by JTM and the findings were crosschecked, first with SB, and additionally by discussions with the whole research team, throughout the research process to define and redefine the codes, categories and themes.

## Results

### Quantitative sub-evaluation. Questionnaires

Of the total 248 training participants enrolled in the CCCT, 192 handed in completed pre-and post-training questionnaires, yielding a response rate of 77%. Descriptive data of participants can be seen in Table 1.

After the training, 77. 6% (*N* = 160) perceived the training to be useful or very useful. Ratings of perceived knowledge pre- and post-training are shown in Table 2. Ratings of perceived knowledge were significantly higher on all items after training. Cronbach’s alpha was α = 0.941 for the pre-training knowledge items and 0.944 for post-training items. See Table 2 for mean values, SD of the pre-and post-items.

We also compared ratings of perceived knowledge pre- and post-training for the subgroups, please see Table 3. Participants who had reported recent experience of receiving refugee patients gave significantly higher ratings in pre-training knowledge, compared to participants that stated no recent experience of refugee patients. Post-training, there was a trend towards significantly higher scores in perceived knowledge among experienced participants. Participants who had stated experience of using interpreters also gave significantly higher scores in perceived knowledge pre-training compared to those without recent experience of using interpreters, while there was no difference between the groups after the training.

**Table 3:** Perceived knowledge: pre- and post-training of sub-groups, t-test independent groups (*N* = 192)

Subgroups	Mean of Perceived knowledge pre- and post-training, total score		T-test				
	Before	SD	After	SD	*t*-value	df	*p*-value
Recent experience of refugee patients (in the past month)	47.21	13.50	62.93	13.44	-2.90	223	0.004
No recent experience of refugee patients (in the past month)	42.11	12.28	59.49	10.56	-1.94	184	0.054
Recent experience of using interpreters (in the past month)	47.36	13.04	61.54	14.05	-3.14	224	0.002
No recent experience of using interpreters (in the past month)	41.91	11.75	61.70	11.01	0.09	186	0.930

**Fig. 2:**
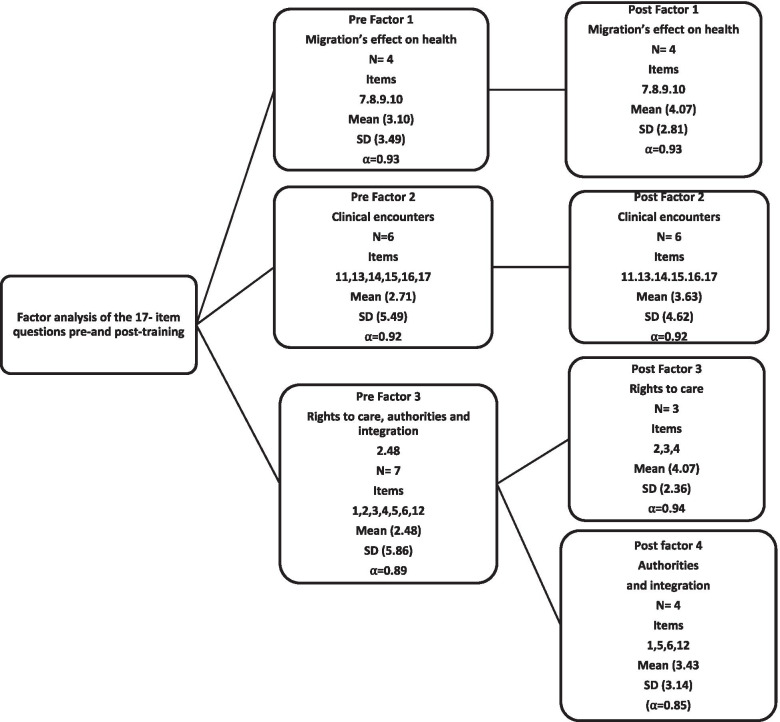
Factor analysis of the item questions pre-and post-training (N = 192)

The factor analysis resulted in 3 factors for the pre-training questionnaire, with eigenvalues over 1.0 explaining 71% of the covariance, and 4 factors for the post-training questionnaire, with eigenvalues over 1.0 explaining 78% of the covariance (see Fig. 2). Descriptions of the items see Table 1.

### Qualitative sub-evaluation. Focus group discussions

Descriptive data of the 28 informants are summarized in Table 4.

**Table 4:** Descriptive data of informants from the FGDs (*N* = 28)

Characteristics		
Organization	Adult psychiatry care	16
	Child and adolescent psychiatry care	12
Occupation/Unemployment	Nurse	3
	Medical doctor	3
	Psychologist	10
	Social workers	5
	Occupational therapist	1
	Auxiliary/psychiatric nurse aide	4
	Management	1
	Consultant	1

Findings from the FGDs are presented in Table 5 in the two-index areas: (1) Perception, experiences and clinical interaction with refugee patients, and (2) training outcomes and implications for service delivery. Most participants responded briefly to the questions regarding content, level, further need of training and their general views of the various parts of the CCCT. However, they discussed the overall knowledge gained and how they would be able to apply it in their clinical work. They gave a wealth of information about their experiences and thoughts regarding encountering refugee and asylum-seeking patients.

**Table 5:** Index areas and themes from focus group discussions performed after training

Index areas	1.Perception, experiences and clinical interaction with refugee patients		2.Training outcomes and implications for service delivery	
Themes	The notion of otherness	Feeling overwhelmed by refugees’ social suffering	Towards better understanding of the complexity of migration and health	Standing up for patients’ rights as a professional
Su-themes	Attitudes towards refugees and refugee health	The social exposure of the other	Migration and its effect on the individual	Standing up for patients’ rights as a professional
	Communication barriers	Not doing well enough	The role of culture in mental health and clinical reasoning	
	Expectations of care	The complexity of several authorities involved	New perspectives promoted empathy	

### Index-area 1: perception, experiences and clinical interaction with refugee patients

#### The notion of otherness

##### Attitudes towards refugees and refugee health

Generally, the informants considered the newly arrived refugees, including undocumented refugees and asylum seekers, as a different and a more challenging group to care for, in comparison with other patient groups. Those informants that had experience of encountering refugee patients, had mostly dealt with crises situations. The patients were from various parts of the world and had diverse cultures, religions, social contexts and migration backgrounds. Further, patients had varying legal status and differing forms of mental ill- health. Main reasons for mental ill-health were considered by the informants to be migration experiences, being newly arrived and experience of trauma. However, some informants said they had not yet encountered patients with a migration background at their current clinic.

*“If you compare them (patients with a refugee background) with other patients… I would say it’s much more challenging, absolutely“ (psychologist)*

*“We haven’t had that sort of patient here…”( social worker)*

In contrast, one informant, with a migration background, described refugee patients in a different manner, she did not understand her colleagues’ dilemmas with the refugee group and said:

*“It doesn’t have to be as difficult as everybody seems to think, it’s a patient group like any other group” (social worker)*

##### Communication barriers

The informants emphasised practical barriers upon encountering refugee patients, where communication challenges were the main problem. The patients’ lack of Swedish language skills and the use of interpreters complicated communication. Informants also indicated that refugee patients’ understanding of mental-ill health was unfamiliar to them, and they faced difficulties in delivering information that patients needed. To have received training and advice on practical solutions of how to work with an interpreter was found very useful.

*“Of course, when you don’t share a common language, it makes it difficult” …(Auxiliary/ Psychiatric nurse aide)*

*“I think the part, “Working with interpreters” (referring to the training) will prove helpful” (medical doctor working in psychiatric care)*

##### Expectations of care

The informants reported that refugees sometimes had expectations that went beyond what could be provided in the clinical setting. Refugee patients were perceived to express mental ill-health through somatic complaints rather than describing their problems as emotional or mental. Additionally, refugees were considered to have a poor understanding and knowledge of the Swedish health care system and to have difficulties in navigating it. The informants also experienced difficulties in explaining mental health care services to refugee patients.

*“Sometimes they don’t even know where they are [referring to the psychiatric services], and one has to explain everything about what we do…"(Management position in psychiatric care) *

#### Feeling overwhelmed by refugees’ social suffering

##### The social exposure of the other

Most of the informants talked about refugee patients’ harsh social and economic situation. They stressed patients’ social insecurity, marginalization, unstable and overcrowded housing, unemployment and difficult family situations. The patients often had to change their accommodation, resulting in difficulties in maintaining a long-lasting patient-caretaker relationship, which in turn affects trust.

*“They (referring to refugee patients) live in very crowded spaces…even with children… they face enormous difficulties in so many ways, even here in Sweden…” (Nurse specialized in psychiatry)*

The informants described how refugees seemed to live in a parallel society, with limited influence on their current living conditions and that they often appeared to be powerless in solving their own social situations. However, some informants suggested that refugees were resourceful and resilient, enabling them to overcome migration challenges. The latter included the ability to manage and adapt to the Swedish culture and context. One informant stressed that, unfortunately, after a time in Sweden, many of the patients experience hopelessness and lose their resilience.

*“… generally, this a very resourceful group…”(Medical doctor specialized in psychiatry)*

*“Somewhere on their journey this resourcefulness breaks down or during the process here [referring to Sweden]…"(psychologist)*

##### Not doing well enough

The informants felt ill-equipped to handle the suffering and social problems of refugees. They also often felt overwhelmed and inadequate regarding their refugee patients’ needs and being responsive to those needs. Some informants described difficulties conducting psychiatric assessments. This was due to patients’ ways of expressing mental-ill health and their exposure to a context of social suffering and distress unfamiliar to the participating professional. The informants experienced that the problems presented to them were often beyond their ability as mental health care professionals to handle and in some cases, even for the health care sectors in general.

*“There is fear or insecurity when working with this group [referring to refugees] … one is worried about the outcome…One doesn’t want to do or say the wrong thing…”(psychologist).*

##### The complexity of several authorities involved

Informants acknowledge the complexity of all the agencies involved in refugee reception and integration. Refugee patients also often have several different contacts in the mental health care system. The informants, as well as refugee patients, are often confused and unaware of all the regulations, the agencies involved and their role.

*“I was surprised how extremely complicated the system is in Sweden…there are so many agencies and stakeholders involved… it’s overwhelming for the patients” (Nurse working in psychiatric care)*

### Index 2. Training outcomes and implications for service delivery

#### Towards better understanding of the complexity of migration and health

##### Migration and its effect on the individual

Most informants expressed that the training provided better understanding of several migration and refugee health-related topics, in particular on the subject of the context and social situations of refugees in Sweden. Moreover, in what way migration experience, trauma and migration stress can affect the health of patients, and their families. The informants also thought that the training contributed to better understanding of the connection and roles of different agencies in refugee reception and how they relate to patients’ situations in Stockholm.

*“I have got a better picture of the complexity of their situations, what they may have gone through…and health aspects…”(psychologist)*

##### The role of culture in mental health and clinical reasoning

Many perceived that after the training they had a better understanding of cultural aspects when encountering patients. Sensitivity to culture was considered to be very important regarding clinical assessment, reasoning, decision making and treatment outcomes. Informants particularly discussed different cultural idioms of expressions of mental ill -health and symptoms and how the training resulted in a greater attentiveness and a sense of being better prepared to approach cultural issues in assessment and encounters.

*” I have a better understanding of how one can express different symptoms … I think there are different ways to view health… that are culturally related…”(Management, position in mental health care)*

*“I think that I have gained a better understanding of how many different things I need to bear in mind in relation to these patients... this is useful for assessment and treatment” (psychologist)*

##### New perspectives promote empathy

For some informants, the training had influenced how they viewed patients with a refugee background, their social suffering, context and circumstances. Informants found that knowledge they gained in the training about various aspects of migration, integration and refugee mental health, contributed to new perspectives. One informant emphasized that the training in general, and some of the lectures in particular, also stimulated empathy. She said:

*“I feel that the whole day has had a very, very respectful tone that I have really missed in mental health care earlier. I think that there is a major attitude problem towards this group of patients. And one really feels that one’s heart bleeds” (nurse working in psychiatric care)*

#### Standing up for patients’ rights as a professional

Informants emphasized that the training promoted reflections over one’s professional role and empowered them in being curious, courageous and standing up for certain values. They felt validated, in the sense of doing the right thing for their patients, despite the circumstances and their work situations.

*“You have to have courage to ask the difficult questions [referring to trauma]… ” (social worker)*

*“I have more of a backbone now, it’s difficult sometimes, but one has to be comfortable” (psychologist)*

*“I feel a certain confirmation, that what I’m doing is good…the right thing. That feels good” (medical doctor)*

## Discussion

This mixed-method evaluation, based on pre-and post-training questionnaires and focus group discussions, aimed to evaluate a comprehensive cross-cultural training program for mental health care professionals in Stockholm. Understanding of the quantitative results was enhanced by qualitative findings, and the FGDs also contributed to findings of their own. Our main findings were that participants experienced gained knowledge and new perspectives in all aspects covered in the CCCT. Further, with improved knowledge, participants restructured their prior knowledge. The FGD informants talked about their views on refugee patients, their emotions of shortcomings and feeling overwhelmed in facing patients’ social suffering, and their professional role. Yet, the informants stated that new knowledge and understanding promoted empathy towards this patient group. Additional qualitative findings highlighted informants’ reflections on a perceived strengthening of their professional role after the CCCT.

The pre-training questions about attitudes revealed that refugee patients were perceived as a more demanding patient group in comparison with other patient groups; and many mental health care professionals had not encountered refugee patients in the past month. The FGDs confirmed that such patients were found more challenging. Participants who had reported experience of refugee patients and working with interpreters, pre-training in the past month, had higher ratings of perceived knowledge. Post-training, there were no significant changes in perceived knowledge between those with experience and those without experience of refugee patients and working with interpreters. This suggests that participants with no recent experience of delivering care to this patient group and working with interpreters seemed to gain most out of the CCCT. These results also indicate that training participants’ up-to date experiences were of significance regarding the effect of the CCCT, and that knowledge was not necessarily based on formal education or training.

Results from the FGDs correspond with other research literature. An improved understanding of diverse cultural idioms of distress may help mental health providers to navigate communication barriers and facilitate proper diagnostic evaluations and treatment outcomes [2, 30]. Other aspects expressed in the FGDs were: self-reflection, awareness of refugees’ background and current situations, making sense of their suffering, and difficulties refugees go through in navigating the Swedish refugee reception system involving many different authorities. The factor analysis showed the dimensionality of the questionnaires, specifically, involving items referring to rights to health care for asylum seekers and undocumented refugees, and additionally, understanding of the role of various authorities involved in refugee reception.

### Restructuring of knowledge

The restructured knowledge post-training was based on the understanding of social responsibilities and obligations regarding refugee health and integration, and the authorities involved. This suggests that participants not only gained more knowledge but also developed a new way of conceiving the domains of knowledge. They also structured their understanding differently post-training. Hence, in the FGDs, the informants reported that although they had acquired knowledge about rights to care and authorities involved in refugee reception, they still found it difficult to understand the complexity of all the regulations and authorities. Thus, more support, understanding and training about social determinants of refugee health, regulations and the role of different authorities involved in refugee integration can provide clearer guidance to mental health professionals. Lack of knowledge among mental health care professionals regarding rights to care for asylum seekers and undocumented refugee patients may create unnecessary barriers to mental health care for these patient groups.

In this evaluation, FGD informants revealed that gained knowledge and new perspectives promoted empathy toward asylum seekers and refugee groups. This is in line with previous studies where increased knowledge among mental health care professionals about asylum seekers and refugees’ situation and suffering was found to promote empathic engagement. It also improved professionals’ capacity for approaching refuges and asylum seekers suffering from mental distress [23, 31, 32]. As emphasized by Rousseau, including societal aspects when training mental health professionals can deconstruct common intolerance of refugees [31].

Finally, our study illustrated that providing training in cross-cultural mental health care can validate and empower mental health professionals. In the FGDs, the informants reported feeling strengthened as care providers and advocates for patients’ rights to care. Improved knowledge among mental health providers about asylum seekers and undocumented refugees’ right to mental health care may reduce barriers and facilitate care for these patient groups.

The CCCT resulted in perceived knowledge development among participants. Findings from this study indicate that cross-cultural training needs to be at an appropriate level, in terms of the participants’ experience of encountering refugees in a clinical setting and their knowledge of refugees’ social situations and suffering in their new setting. Cross-cultural training also benefits an understanding of cultural aspects and includes reflexivity about emotional aspects. Training and providing working models for mental health professionals in cross-cultural issues may facilitate improved quality of care for refugee groups, reduce barriers [31] and strengthen training participants in their professional role.

### Strengths and limitations

This mixed-method evaluation has several limitations. First, we did not follow-up long-term effects and participants’ experiences of usefulness in their clinical work after having completed the training, which would have been valuable. However, due to the diversity of organizations and care providers that participated in the CCCT, it would have been very difficult to reach the participants for a follow-up survey. At this point, we cannot say what effect the training has had on their professional clinical practices. Previous evaluation of cross-cultural training programs in Region Stockholm showed difficulties conducting a follow-up survey due to the limited possibility of participants being absent from clinical work [24].

Second, of all the introductory CCCT participants, 56 (19.7%) were lost to follow-up in the pre- and post-training questionnaires. The reason for this may be that on two occasions, due to logistic clinical challenges and situations, we reduced the CCCT and conducted two half-days instead of two one-day training sessions. Therefore, the quantitative results in this study were based on 192 pre-and post-training questionnaires.

Third, self- selection bias in that all mental health professionals in Stockholm were eligible to enroll. Although all participants chose to participate by signing up for the training, based on interest and need, the CCCT included a mixed group of mental health professionals with different occupations, education levels and from various organizations. Our training program allowed them the opportunity to exchange experiences.

Fourth, since we only included mental health professionals in our training program, we cannot say how the findings apply to other health professionals.

One strength of this evaluation was the use of a mixed method approach, with an embedded design, allowing for qualitative data to shed light on the quantitative data, and providing opportunities to investigate the cross-cultural training from various perspectives. Additional strengths were that one researcher collected all the data and the use of heterogeneous purposive sampling, which involved various mental health professional groups.

The first author, JTM, is a public health professional with a migration background. JTM moderated all FGDs and may have influenced the overall discussions of cross-cultural matters and the approach of how participants spoke about refugees and their rights to access mental health care. Her insider-outsider perspective on refugee experiences possibly affected the discussion about refugees in the FGDs by, in some way, being linked to the group. JTM has previous experience in conducting qualitative research in cross-cultural contexts and is trained in qualitative research methodology.

### Implications

For cross-cultural training of mental health professionals, this evaluation proposes that training should address cognitive, cultural and social, as well as emotional aspects of encountering refugees, asylum seekers and undocumented refugees with mental ill-health in a context of social suffering. Cross-cultural training of mental health professionals may assist the progress of improved quality of care and reduce barriers to mental health care for asylum seekers, undocumented refugees, and refugee groups.

## Conclusion

The present evaluation showed that one-day cross-cultural training contributed to perceived knowledge development and attitude changes among participating professionals. The training participants felt strengthened in their professional role in encountering refugee patients. An additional outcome was that participants restructured their prior knowledge towards new understandings of social responsibilities and obligations regarding refugee health, integration and authorities. Further, the qualitative part of this mixed-method evaluation revealed the professionals’ experiences of feeling overwhelmed by refugee patients’ social predicament of suffering and that this affected both clinical encounter and treatment.

## Supplementary Information

**Additional file 1.**

## Data Availability

The quantitative dataset generated and analysed during the current study is available from the corresponding author on request. The qualitative dataset generated and analysed during the current evaluation is not publicly available due to protection of participants’ anonymity and confidentiality.

## Abbreviations

CCCT: Comprehensive Cross-Cultural Training
FGD: Focus Group Discussion
TC: Transcultural Centre
TCA: Thematic Content Analysis
