# Supplementary material for: Cross-cultural training program on mental health care for refugees - a mixed method evaluation
Source: BMC Med Educ. 2021 Oct 15;21:533. doi: 10.1186/s12909-021-02965-5 (PMC8520228; doi:10.1186/s12909-021-02965-5)
Supplement: Supplementary file 1 — Additional file 1. [file 12909_2021_2965_MOESM1_ESM.docx]

**The FGD interview guide**

**Experience of meeting asylum seekers and refugees**

In your work, is it significant whether the patient is undocumented, an asylum seeker or a refugee who has recently received a residence permit? What is the significance?
In what situations do you encounter this patient group?
What experiences do you have of this patient group?

**Attitudes and perceptions about the introductory CCC- training**How did you experience the training? Structure? Content?
Was the educational information new to you? Something that surprised you?

How has the training affected your understanding of this patient group?
How has the training affected your understanding of the patient group's health?

**Knowledge and skills to meet refugee patients after the CCC-training**What did you find the most useful?

**Supplementary questions:**In what way has the training led to any help in your clinical work?
Do you experience an increased interest in in-depth knowledge about migration and trauma at your workplace after the training?
What do you think about the educational needs at your workplace?
How could one meet these needs?
What was lacking in the training?
Suggestions for improvements?

What do you need more knowledge about?
